# MicroRNA regulation of tumorigenesis, cancer progression and interpatient heterogeneity: towards clinical use

**DOI:** 10.1186/s13059-014-0445-8

**Published:** 2014-08-31

**Authors:** S Patrick Nana-Sinkam, Carlo M Croce

**Affiliations:** Division of Pulmonary, Allergy, Critical Care and Sleep Medicine, The Ohio State University, 473 West 12th Avenue, Columbus, Ohio 43210 USA; James Comprehensive Cancer Center, The Ohio State University, 300 West 10th Avenue, Columbus, Ohio 43210 USA; Department of Molecular Virology, Immunology & Medical Genetics, The Ohio State University, 410 West 10th Avenue, Columbus, Ohio 43210 USA

## Abstract

In the past two decades, microRNAs have emerged as crucial mediators of organ development and human disease. Here, we discuss their role as drivers or suppressors of the hallmarks of cancer during tumorigenesis and progression, in defining interpatient heterogeneity and the promise of therapeutic application.

## Introduction

Cancer continues to generate a significant global medical and socioeconomic burden, despite molecular-based screening, targeted therapeutics, and our increased understanding of the molecular pathogenesis of tumor initiation and progression. As we move into an era of personalized cancer management, an increased understanding of cancer risk and tumor heterogeneity will be essential to the success of early detection and new targeted therapies. High-throughput platforms designed to interrogate the human genome and proteome are successfully being used to improve our understanding of the heterogeneity that exists within both solid and hematological malignancies, and to develop clinical biomarkers and discover new therapeutic targets.

**Figure 1:**
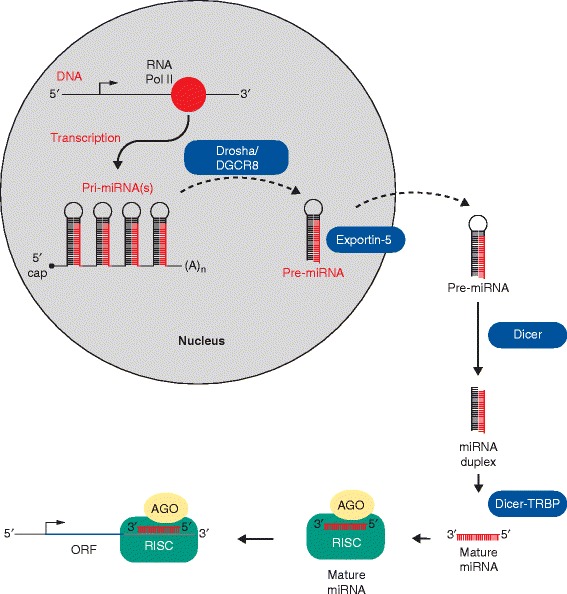
**MicroRNA processing.** During microRNA (miRNA) biogenesis, a primary miRNA transcript (pri-miRNA) is generated by an RNA polymerase (Pol) II (or III, not shown). This is followed by cleavage of the pri-miRNA by the microprocessor complex Drosha-DGCR8 (Pasha). This results in generation of the pre-miRNA, which is then exported from the nucleus by Exportin-5-Ran-GTP. Once in the cytoplasm, the RNase Dicer induces cleavage of the pre-miRNA hairpin to a double stranded mature length. The functional strand of the mature miRNA is loaded together with Argonaute (Ago2) proteins into the RNA-induced silencing complex (RISC). This complex then guides the miRNA to target mRNAs for mRNA cleavage, translational repression or deadenylation. ORF, open reading frame; TRBP, trans-activation response RNA-binding protein.

In the past two decades, investigators have identified non-coding components of the human genome, such as microRNAs (miRNAs), as critical mediators of organ development and human disease [1]. miRNAs are non-coding RNAs 18–25 nucleotides in length that are endogenously processed in the cell and then target RNA for silencing either by RNA degradation or inhibition of transcription (Figure 1) [2]. Given the fact that miRNAs have the capacity to regulate tens to hundreds of mRNAs, this can result in the simultaneous regulation of multiple biological pathways [3,4]. miRNAs tend to be localized to fragile chromosomal regions that are susceptible to deletions, translocations and amplifications [5]. These same chromosomal regions are often altered in cancers. Additionally, miRNAs may exist within introns or exons (harboring an independent promoter) or within a host gene [6]. Furthermore, the mechanisms by which epigenetic, genetic and environmental factors converge to regulate miRNAs in cancer are also complex and continue to be uncovered.

The applications of miRNAs in the clinical management of cancers are starting to be realized, as evidenced by an increasing number of miRNA-based clinical trials. Specifically, miRNAs are being applied as both prognostic and therapeutic biomarkers in the setting of clinical trials. The goal is to use miRNAs as another means for delineating the molecular and clinical heterogeneity that exist within cancers (Table 1). As we increasingly apply high-throughput strategies, such as genome sequencing, and identify additional oncogenic mutations that drive tumorigenesis and define heterogeneity, it is likely that a larger role for miRNAs in cancer biology will be uncovered.

**Table 1:** **Selected microRNA-based clinical trials**

**Cancer type**	**Type of trial**	**Intervention or tissue type**	**Clinical application**	**Reference**
Hepatocellular or liver metastasis	Therapeutic	miR‐34 delivery (MRK-34)	Phase 1 toxicity trial	[7]
Thyroid	Biomarker	Tissue (fine needle aspiration)	Diagnosis, chemoresponse	[8]
Breast	Biomarker	Blood	Prognosis, surveillance, cardiotoxicity	[9]
Breast	Biomarker	Tissue	Chemoresponse, diagnosis	[9]
Glioma	Biomarker	Tissue	miR-10b levels in diagnosis and prognosis	[10]
Leukemia	Biomarker	Blood	miR-155 as predictor of graft versus host disease	[11]
Lung	Biomarker	Tissue	Chemoresponse	
Neuroblastoma	Biomarker	Tissue	Prognosis, tumor classification	[12]
Colon	Biomarker	Tissue	Disease stratification, chemoresponse	[13]
Renal	Biomarker	Cell	Tissue chemoresponse	[14]
Esophageal	Biomarker	Tissue	Predictor of response to zinc	
Melanoma	Biomarker	Blood	Predictor of response to propranolol	[15]

miR, microRNA.

The early observations that miRNAs are globally dysregulated in cancer suggested that miRNAs may contribute to both the initiation and progression of cancers [16–18]. Since these initial observations, miRNAs have been implicated as either effectors or targets of almost all of the hallmarks of cancer, including proliferation, resisting cell death, avoiding growth suppression, angiogenesis, replicative immortality and invasion/metastasis [19–23]. The roles of miRNAs as key regulators of each of these hallmarks of cancer continue to be extensively investigated and have been previously reviewed [24]. As investigators continue to identify new mechanisms for cancer initiation and progression, and to elucidate the relative contributions of the primary tumor and components of its environment to carcinogenesis, the eventual application of miRNAs in human cancer will come to fruition. For example, the recent observation that miRNAs may be integral to reprogramming of energy metabolism in cancer has provided an additional layer of complexity to miRNAs in cancer and led to the identification of new targets.

Here, rather than attempt to review the broad field of miRNAs in cancer biology, we provide an overview of some of the most promising and emerging areas in miRNA investigation. In particular, we focus on miRNAs as regulators of metabolic programming, their role in metastasis, the new concept of extracellular vesicular miRNAs as drivers of tumorigenesis and, lastly, the biological links between miRNAs and established oncogenic mutations. Each of these new and exciting areas has the potential for translating to clinical application.

### MicroRNAs and metabolic reprogramming in cancer

The contribution of metabolic reprogramming of cancer cells to a largely glycolytic pathway to tumor initiation and progression remains poorly understood. However, there is increasing evidence that tumor dependence on anaerobic glycolysis regardless of oxygen stores (known as the Warburg effect) is driven by both tumor suppressors and oncogenes [25,26]. This metabolic shift confers a biological advantage to cancer cells, thus supporting both initiation and progression of tumors. Several miRNAs have been shown to regulate cancer cell metabolism, either through direct regulation of components of glycolysis or by targeting key oncogenic pathways.

The discovery that the miR-15a/16/1 cluster is downregulated or deleted in chronic lymphocytic leukemia was one of the first observations of miRNA deregulation in cancer [22]. This initial observation further supported the concept of miRNAs being localized to fragile chromosomal regions. We identified both aldolase A (*ALDOA*) and triosephosphate isomerase 1 (*TPI1*), which have been both implicated in glycolysis, as potential targets for miR-15a/16-1. ALDOA is central to the glycolytic process and its overexpression in lung cancer has been associated with poor survival [27]. Since these initial observations, several other miRNAs have been identified as regulators of components of metabolic pathways in cancer. For example, monocarboxylate transporters (MCTs) contribute to lactate homeostasis through both influx and efflux across cell membranes. As a result, targeting MCTs is a viable therapeutic approach in cancer. Several tumor suppressor miRNAs, including miR-29b, have been shown to target components of lactate generation, including MCTs [28]. A third example of miRNA-mediated regulation of cellular metabolism involves one of the earliest described miRNAs. First described in *Caenorhabditis elegans* as crucial to larval development [29], Lin-28 is a highly conserved RNA-binding protein that has been shown to regulate glycolytic enzymes, components of mitochondrial oxidative phosphorylation and the miRNA Let-7 [29]. In addition, Lin-28 has emerged as an oncogene [30]. A recent study showed that Lin-28a is essential to programming bioenergetics during embryonal development and that Lin-28a-driven reprogramming of metabolism contributes to tissue repair [31]. The potential links between Lin-28 and cancer metabolism are still being investigated. Lastly, Singh and colleagues [32] recently identified a new link between miRNA deregulation, cancer metabolism and tumor progression. The authors [32] observed that the redox-sensitive basic leucine zipper family transcription factor nuclear factor erythroid-2-related factor 2 promotes tumorigenesis both *in vitro* and in a murine model through epigenetic regulation of miR-1 and miR-206. This leads to reprogramming of glucose metabolism through activation of components of the pentose phosphate pathway. The relationships between miR-1, miR-206 and components of the pentose phosphate pathway were also observed in human tissues [32].

The biological relationships between miRNAs and components of cellular metabolism continue to be established particularly in the setting of tumorigenesis. These findings will further serve to drive our understanding of the biology of cancer metabolism in tumor progression miRNAs as potential therapeutic targets in altering these important pathways.

### MicroRNAs and metastasis

Metastatic disease is a major cause of mortality in solid malignancies. For instance, in lung cancer, less than 20% of patients present with localized disease. Thus, the identification of actionable targets that can be used clinically both as biomarkers and as potential therapeutic targets in the metastatic setting is essential. The process by which tumors progress to metastatic disease is a complex one. This process relies on a sequence of events that integrate both cues from the primary tumor and the surrounding microenvironment. Epithelial-mesenchymal transition (EMT) is a central mechanism that drives the eventual detachment, invasion, circulation and extravasation of cancer cells that define the metastatic process [33]. miRNAs have been shown to regulate the metastatic process by either targeting components of EMT, targeting signaling pathways that drive EMT or by selective targeting of other miRNAs. Given that miRNAs are increasingly being used as both directed therapies and as diagnostic/prognostic biomarkers, one could envision select miRNAs being targeted in cases of metastatic disease or being used as biomarkers to monitor tumor response or recurrence.

The miR-200 family is perhaps the best-studied group of miRNAs that are known to target key transcriptional drivers of EMT, such as ZEB-1 and ZEB-2 [34–37]. For example, Davalos *et al.* [38] showed that miR-200 is susceptible to dynamic methylation silencing in cancer cells with mesenchymal properties but is unmethylated in tumors maintaining epithelial characteristics. In addition, tumor-suppressive miRNAs are also susceptible to regulation by mediators of EMT during metastasis. For example, Yang and colleagues [39] determined in a murine model of *Kras*-induced lung adenocarcinoma that a subpopulation of metastasis-prone cancer cells were dependent on Jagged2 for their metastatic potential both *in vitro* and *in vivo.* Furthermore, they determined that Jagged2 drives metastasis through the downregulation of miR-200 [39]. A separate study [40] showed that members of the miR-200 family are not the only miRNAs implicated in the metastatic process. ZEB1 promotes metastasis through the downregulation of the tumor suppressor miR-34a [40]. The observation that miRNAs may either drive or suppress hallmarks of cancer through direct or indirect regulation of other miRNAs is a relatively new one that has emerged in the past few years. For example, this concept has been observed in promoting the metastatic process. A recent study by Song *et al.* [41] demonstrated that miR-22 could contribute to and promote metastases in breast cancer through miRNA regulation. The investigators showed both *in vitro* and *in vivo* that miR-22 can suppress miR-200 through direct targeting of members of the Ten eleven translocation family, thus leading to hypermethylation of members of the miR-200 family [38].

The process of metastasis is a complex one that currently lacks any therapeutic target. As a result, metastatic cancers can be extremely challenging to treat. The increasing number of studies linking miRNAs, particularly members of the miR-200 family, to components of EMT has increased our understanding of the biology of EMT and the potential for miRNAs as regulators of EMT. Although these studies have yet to translate to clinical application, they do provide the opportunity for using miRNAs as biomarkers of therapeutic response in cases of advanced disease or in the setting of surveillance.

### Extracellular microRNAs as drivers of tumorigenesis

One particular area that has evaded investigators has been the process by which miRNAs may drive local and distant tumor-environment interactions. Recently, studies have shown that miRNAs may circulate either freely packaged within extracellular vesicles (EVs) or bound to specific proteins (such as the miRNA processing molecule Ago2) [42-44]. EVs are a family of small membrane-encapsulated fluid particles, comprising shedding microvesicles and exosomes, that are released from either multivesicular bodies or directly from plasma membranes [45]. EVs are released from a wide variety of cell types by independent cellular mechanisms and have become the focus of intense investigation [45,46]. Studies have shown that, in cancers, both tumor- and stromal-derived EVs (and their contents, including miRNAs) may function as conduits for the transport and release of mediators that drive or suppress the immune response, angiogenesis, inflammation, extracellular matrix remodeling and invasion/metastasis [47-50]. EVs 'communicate' with cells by fusing to the plasma membrane and delivering their functional cargo of proteins, lipids, mRNAs or miRNAs.

We recently identified an additional biological function for exosomal miRNAs [51]. We demonstrated [51] that exosomes may serve as ligands for Toll-like receptor-8 (TLR-8) in humans and its homolog Tlr7 in mice. We determined that certain miRNAs, including miR-21 and miR-29, compartmentalized within exosomes could activate the TLR-8 in a recipient cell to activate an inflammatory pro-metastatic response involving nuclear factor κB and the secretion of tumor necrosis factor alpha and interleukin-6 [51]. These observations suggested an additional paracrine function for miRNA in driving the metastatic process that could be targeted.

Exosomes and microvesicles, which range in size from 30 to 1,000 nm, are detectable in several body fluids ranging from plasma to urine to sputum and are the likely explanation for the observed stability of miRNAs in body fluids [52]. As a result, they are being investigated as potential non-invasive biomarkers in several cancers. Early studies [53–56] demonstrated that circulating exosomes could be used as potential diagnostic tools in ovarian, lung and prostate cancers.

Our understanding of EVs in tumorigenesis remains in its infancy; however, several studies now suggest that they may be central to intercellular communication in cancer. Additionally, their discovery supports a new area of investigation of EVs as non-invasive biomarkers of disease. Lastly, an interesting but relatively unexplored therapeutic strategy for cancer involves the directed targeting of EVs.

## MicroRNAs in cancer heterogeneity

High-throughput analyses of the human genome have led to the discovery of several previously unrecognized somatic mutations that contribute to the development of both solid and hematological malignancies. In several cancers, including lung cancer, assessment of patients for specific, more common mutations has become standard of care to guide the use of therapies that target those mutations; however, several relatively common mutations in cancer remain without effective therapies. For patients with these mutations in particular, miRNAs that function as effectors, targets and predictive biomarkers in mutation-driven cancers can provide us with additional insight into the underlying mechanisms involved and help guide drug development and therapeutic decisions. Below, we provide some examples of more common mutations that are being applied in the clinical setting.

### Kras

Approximately 20-25% of tumors harbor mutations in genes encoding Ras family proteins, with Kras being the most common. Through activation of downstream molecules, including the Raf-MEK-ERK and PI3K-Akt pathways, Kras signaling confers both a growth and survival advantage to tumors. Although many Kras-targeted therapeutics are in clinical trials, none have yet been approved for use in the clinic. The primary reason for the lack of therapeutic targets in Kras-driven cancers is the recognized complexity of Kras signaling. Thus, it is imperative that investigators continue to identify new actionable pathways that may be eventually applied in the large percentage of cancers that harbor these mutations.

Several miRNAs have been found to regulate *Kras*, including Let-7, one of the earliest described tumor suppressor miRNAs that has been shown to be downregulated in cancers [57]. This initial observation has led several researchers to identify polymorphisms within the Let-7-complementary *Kras* 3′ untranslated regions [58–61] and Let-7 expression as prognostic biomarkers in cancer. Furthermore, given the establishment of Let-7 as a tumor suppressor, Let-7 has been systemically delivered *in vivo* as a directed therapeutic in lung cancer [62,63]. Two additional miRNAs, miR-30b and miR-27, have been also validated as direct regulators of *Kras* and function as tumor suppressors in solid cancers [64,65]. A separate study demonstrated that use of a miR-34 mimic could function as a tumor suppressor and be applied as a directed therapeutic in a murine model of Kras-induced lung tumorigenesis [66]. Conversely, the inhibition of miRNAs such as miR-143 and miR-145 by oncogenic Ras signaling has been identified as a mechanism for driving tumor progression [67]. The miRNA miR-21 is consistently deregulated in cancers, including lung, glioblastoma and B-cell lymphoma [68–70], and Hatley *et al.* [71] demonstrated that miR-21 is a potent driver of lung tumorigenesis in a murine model of Kras-induced lung adenocarcinoma. They showed that miR-21 directly antagonizes inhibitors of Ras, including sprouty1, sprouty2, B-cell translocation gene 2 and programmed cell death 4 [71].

### Epidermal growth factor receptor

The discovery of epidermal growth factor receptor (*EGFR*) mutations in lung cancer was a major step forward, as was the successful targeting of *EGFR*-mutated tumors using EGFR tyrosine kinase inhibitors (TKIs such as gefitinib or erlotinib), which enables individualized therapy in approximately 10% of patients with lung cancer [72]. Interestingly, miRNAs may both drive EGFR signaling and serve as targets for EGFR signaling.

A recent elegant study by Shen and colleagues [73] determined that Ago2 could function as a binding partner for EGFR. In particular, in response to hypoxia, EGFR directly phosphorylates Ago2 at Tyr393, thus interfering with precursor miRNA loading and processing. Other studies have attempted to correlate *EGFR* mutational status with patterns of miRNA expression. Investigators have also attempted to use miRNAs as *EGFR*-specific diagnostic biomarkers. For example, Bjaanaes *et al.* [74] identified 17 miRNAs, including miR-184 and miR-30d, that distinguished *EGFR*-mutated lung adenocarcinomas from wild-type tumors. Additionally, they identified three miRNAs that distinguished *Kras*-mutated lung cancers from wild-type tumors; however, the biological significance of most of these miRNAs and their validation as *Kras*-specific biomarkers remains relatively unexplored [74]. In an independent investigation, miR-21 levels were identified as a predictor of response to EGFR TKI therapy [75].

For clinicians, managing the development of EGFR inhibitor resistance in patients continues to be a major obstacle. Although several mechanisms for intrinsic resistance to EGFR inhibitors have been identified, a substantial percentage of resistance develops with no known mechanism. Investigators have demonstrated that particular miRNAs may function as actionable targets in managing resistance. Park *et al.* [76] discovered that CRIPTO-mediated (an EGF-CFC protein family member involved in transforming growth factor β signaling) downregulation of miR-205, which leads to EMT and activation of the Src oncogene pathway, is a mechanism for EGFR inhibitor resistance in non-small-cell lung cancer. Re-expression of miR-205 led to downregulation of EMT and Src activation, and restored EGFR-inhibitor sensitivity, suggesting that miR-205 may serve as a predictive biomarker of response to EGFR inhibition [76]. Another example in which it would be useful to predict which patients would benefit from EGFR antibody therapy is metastatic colorectal cancer. Although targeting EGFR using monoclonal antibodies, such as cetuximab and panitumumab, has proven successful in metastatic colorectal carcinoma, less than half of patients with *Kras* wild-type tumors are responsive to EGFR targeting. miR-31-3p expression was identified as a predictive biomarker in colorectal cancer patients receiving EGFR antibody therapy [77].

### Liver kinase B1

In the late 1990s, investigators discovered that a germline inactivating mutation in the serine threonine kinase liver kinase B1 (*LKB1*) gene increased susceptibility to Peutz-Jeghers syndrome, which manifests as sporadic benign and malignant tumors in several organs [78]. LKB1 activates several downstream kinases, including AMP-activated kinase, which has a role in cellular energy homeostasis. In energy-deficient states in particular, LKB1 functions as a tumor suppressor [79]. Several studies have since shown that somatic mutations leading to LKB1 inactivation may contribute to the pathogenesis of malignancies, including breast, liver, colorectal and lung, by metabolic reprogramming of the cancer cell and conferring a growth and metastatic advantage [80]. The role of miRNAs in LKB1 signaling in cancer remains poorly understood. The few studies so far have been conducted in gliomas. This gap in knowledge is of particular importance given the increasing awareness of the importance of LKB1 inactivation in solid tumors as an additional subset of cancers requiring targeted therapeutics.

In one of the earliest investigations of LKB1, Godlewski and colleagues [81] determined that miR-451 was overexpressed in gliomas and correlated with poor survival. They found that miR-451 targeted CAB39, a key component of the LKB1 complex. The investigators concluded that in normal glycemic conditions elevated miR-451 suppressed LKB1 signaling, leading to a proliferative advantage in glioma cells. Conversely, in conditions of metabolic stress, such as low glycemic states, miR-451 is decreased, and the resultant increase in LKB1 signaling promotes cell survival and motility. miRNAs may also indirectly regulate LKB1 signaling, as evidenced by the observation that miR-204 downregulation promotes migration and invasion through upregulation of Sirtuin1 (SIRT1), which leads to downregulation of LKB1 [82].

### Anaplastic lymphoid kinase

Deregulation of anaplastic lymphoid kinase (ALK), a tyrosine kinase first described as part of an oncogenic fusion protein in anaplastic large-cell lymphoma (ALCL), has emerged as a biomarker that can inform therapeutic decision-making in cancer [83,84]. Perhaps one of the best examples is the identification and selection of sub-populations of lung cancer patients (between 2-7%) harboring molecular rearrangements in *ALK* who have demonstrated survival benefit with the use of ALK-targeting agents such as crizotinib [85].

Knowledge of the links between ALK biology and miRNA is growing, with several studies identifying distinct patterns of miRNA expression in *ALK*^+^ versus *ALK*^−^ malignancies, including ALCL [86]. Most of these studies have focused on ALCL. Liu *et al.* [87] recently identified a panel of seven miRNAs that distinguished *ALK*^+^ from *ALK*^−^ ALCLs (miR-512-3p, miR-886-5p, miR-886-3p, miR-708, miR-135b, miR-146a and miR-155), although the biological consequences of these patterns remain largely unknown. However, two recent studies in ALCL identified downregulated miR-29 as an inducer of cell survival through targeting of induced myeloid leukemia cell differentiation protein (MCL-1) [88] and downregulated miR-16 as a driver of vascular endothelial growth factor expression in *ALK*^+^ disease [89]. Additionally, the c-Myc-driven cluster miR-17-92 has been shown to serve as a mediator of signal transducer and activator of transcription signaling in *ALK*^+^ ALCL [90], thus supporting a role for miRNAs in the biology of ALK signaling.

## Conclusions

In the past two decades, miRNAs have emerged as molecules central to cancer biology. miRNAs represent an additional layer of complexity to cancer biology and have been further validated as regulators of processes fundamental to cancer. The rapid expansion in knowledge regarding the molecular underpinnings of cancer initiation and progression and identification of new somatic mutations has only served to expand the landscape of cancer biology. miRNAs clearly have an integral role in driving cancer. This is a field that is rapidly changing, with metabolic reprogramming and extracellular vesicular biology representing two of the most recent potential actionable targets.

Here, we have reviewed emerging concepts in miRNAs as drivers of cancer heterogeneity and progression with a focus on metabolic reprogramming, metastasis and extracellular vesicular biology. Over the past 10 years, we have moved from a phase of recognition of the global deregulation of miRNAs in cancer to one of validation of miRNAs as tumor suppressors or oncogenes, and we are moving to a future that includes clinical trials with miRNAs as therapeutic biomarkers and as directed therapies. For example, miRNAs including miR-122 and miR-34 are currently in clinical trials as directed therapies in hepatitis C and hepatocellular carcinoma, respectively. There remain several obstacles to miRNAs reaching their full clinical application. These include improving our understanding of their fundamental biology in regulating biological pathways, developing improved methods for their reliable detection and the development of new and safe methods for *in vivo* delivery. Although miRNAs have yet to reach clinical practice, they are poised to do so in the near future.

## Abbreviations

ALCL: anaplastic large-cell lymphoma
ALK: anaplastic lymphoid kinase
EGFR: epidermal growth factor receptor
EMT: epithelial-mesenchymal transition
EV: extracellular vesicle
LKB1: liver kinase B1
MCT: monocarboxylate transporter
miRNA: microRNA
TKI: tyrosine kinase inhibitor
TLR: Toll-like receptor
